# Delineation of intracavitary electrograms for the automatic quantification of decrement-evoked potentials in the coronary sinus with deep-learning techniques

**DOI:** 10.3389/fphys.2024.1331852

**Published:** 2024-05-07

**Authors:** Guillermo Jimenez-Perez, Juan Acosta, Álvaro J. Bocanegra-Pérez, Eduardo Arana-Rueda, Manuel Frutos-López, Juan A. Sánchez-Brotons, Helena Llamas-Gómez, Rodrigo Di Massa Pezzutti, Carmen González de la Portilla Concha, Oscar Camara, Alonso Pedrote

**Affiliations:** ^1^ PhySense Research Group, BCN MedTech, Universitat Pompeu Fabra, Barcelona, Spain; ^2^ Arrhythmia Unit, Department of Cardiology at Virgen Del Rocío University Hospital, Sevilla, Spain

**Keywords:** intracavitary electrograms, decrement-evoked potentials, deep-learning, automatic signal delineation, coronary sinus, local field components, synthetic data

## Abstract

Cardiac arrhythmias cause depolarization waves to conduct unevenly on the myocardial surface, potentially delaying local components with respect to a previous beat when stimulated at faster frequencies. Despite the diagnostic value of localizing the distinct local electrocardiogram (EGM) components for identifying regions with decrement-evoked potentials (DEEPs), current software solutions do not perform automatic signal quantification. Electrophysiologists must manually measure distances on the EGM signals to assess the existence of DEEPs during pacing or extra-stimuli protocols. In this work, we present a deep learning (DL)-based algorithm to identify decrement in atrial components (measured in the coronary sinus) with respect to their ventricular counterparts from EGM signals, for disambiguating between accessory pathways (APs) and atrioventricular re-entrant tachycardias (AVRTs). Several U-Net and W-Net neural networks with different configurations were trained on a private dataset of signals from the coronary sinus (312 EGM recordings from 77 patients who underwent AP or AVRT ablation). A second, separate dataset was annotated for clinical validation, with clinical labels associated to EGM fragments in which decremental conduction was elucidated. To alleviate data scarcity, a synthetic data augmentation method was developed for generating EGM recordings. Moreover, two novel loss functions were developed to minimize false negatives and delineation errors. Finally, the addition of self-attention mechanisms and their effect on model performance was explored. The best performing model was a W-Net model with 6 levels, optimized solely with the Dice loss. The model obtained precisions of 91.28%, 77.78% and of 100.0%, and recalls of 94.86%, 95.25% and 100.0% for localizing local field, far field activations, and extra-stimuli, respectively. The clinical validation model demonstrated good overall agreement with respect to the evaluation of decremental properties. When compared to the criteria of electrophysiologists, the automatic exclusion step reached a sensitivity of 87.06% and a specificity of 97.03%. Out of the non-excluded signals, a sensitivity of 96.77% and a specificity of 95.24% was obtained for classifying them into decremental and non-decremental potentials. Current results show great promise while being, to the best of our knowledge, the first tool in the literature allowing the delineation of all local components present in an EGM recording. This is of capital importance at advancing processing for cardiac electrophysiological procedures and reducing intervention times, as many diagnosis procedures are performed by comparing segments or late potentials in subsequent cardiac cycles.

## 1 Introduction

Understanding deviations in electrical conduction patterns is a key task when diagnosing cardiac arrhythmias (CAs) in electrophysiology (EP) procedures Porta-Sánchez et al. (2018). During EP interventions, a series of local activation patterns or electrograms (EGM) are recorded, which correspond to depolarization waves captured by special catheters. While these EGMs are represented as isolated electrical deflections in normal cardiac tissue, CAs cause depolarization waves to conduct unevenly on the myocardial surface, which alter the morphology of an EGM, induce decremental response of the tissue, generate fractionations in the local components (local fields, LF) or produce the appearance of late potentials (LP) Zeppenfeld and Porta-Sánchez (2020).

Decremental response is especially important as a diagnostic marker. Decrement occurs when local components are delayed with respect to a previous beat when stimulated at faster frequencies. This decrement may be naturally caused (e.g., the AV node delays conduction at faster firing frequencies) or induced by lesions in the myocardium. Current clinical guidelines hint at the diagnostic value of decrement-evoked potentials (DEEPs), which are portions of tissue presenting decremental conduction. Those DEEPs are diagnosed by producing extrastimuli in specific myocardial positions Acosta et al. (2016), Acosta et al. (2020). In this work, the presence or absence of decrement in atrial components (measured in the coronary sinus, CS) with respect to their ventricular counterparts will be explored for disambiguating between accessory pathways (APs) and atrioventricular reentrant tachycardias (AVRTs).

Despite the importance of localizing the distinct local EGM components for assessing the existence of DEEPs, current software solutions do not perform automatic signal quantification Zeppenfeld and Porta-Sánchez (2020). Electrophysiologists must manually measure distances on the EGM signals to assess the existence of DEEPs during pacing or extrastimuli protocols. Even state-of-the-art 3D electroanatomical mapping systems (EAMs) only locate the local field signal with the largest deflection within a cardiac cycle Zeppenfeld and Porta-Sánchez (2020) with relatively simple and error-prone algorithms, which often forces EAM operators to reassign fiducials Zeppenfeld and Porta-Sánchez (2020).

Some computational solutions for EGM signal analysis exist. These algorithms are based on calculating digital signal processing (DSP)-based transformations on the data, such as filtering or Fourier/wavelet transforms (FT and WT, respectively), which aid in reducing data complexity for producing robust signal detection. Osorio et al. (2017) produced an algorithm based on filtering out high-frequency components for locating local components in AF recordings. Similarly, Felix et al. (2015) used a threshold-based WT pipeline for estimating LFs. In Faes et al. (2002), the authors proposed to estimate the local activation time (LAT) from the barycenter of LFs in bipolar EGMs, after filtering and adaptive thresholding. On the other hand, Hajimolahoseini et al. (2018) used a Gaussian mixture model for the analysis of the natural logarithm of the signal. To the best of our knowledge, only Alcaine et al. (2013), Alcaine et al. (2014) directly attempted EGM delineation. The authors firstly delineated onsets and offsets of the surface QRS complex, which was used for windowing the EGM. Then, the WT was used on the signal’s envelope alongside a rule-based algorithm to determine the onset/offset pair of the LFs, reaching good delineation performance. This approach, however, cannot be used to delineate isolated LPs or extra LFs in patients with AF, preventing its usage as a general purpose tool. Neither of the aforementioned works in the literature produce detections of individual waves outside the most salient component, with only Alcaine et al. (2013), Alcaine et al. (2014) computing the onsets and offsets of the predicted wave.

In recent times, deep learning (DL) algorithms have gained popularity for automated data analysis, given their minimal pre-processing requirements and high performance. In the specific case of cardiac signals, some solutions exist for automatic electrocardiogram (ECG) quantification Jimenez-Perez et al. (2019), Jimenez-Perez et al. (2021a), Jimenez-Perez et al. (2021b). However, not many algorithms have been developed for analyzing EGMs, and they revolve around classification Rodrigo et al. (2021). In this work, several fully-convolutional network (FCN), the U-Net Ronneberger et al. (2015) and the W-Net Xia and Kulis (2017) with different configurations, were trained on a private dataset of signals from the CS. To alleviate data scarcity, a synthetic data augmentation method was developed for generating EGM recordings. Moreover, two novel loss functions were developed to minimize false negatives and delineation errors. Finally, the addition of self-attention mechanisms and their effect on model performance was explored Wang et al. (2020). To the best of our knowledge, this is the first developed approach for delineation of intracavitary electrocardiograms (iECG), bridging the gap between the ECG and iECG communities.

## 2 Materials and methods

This section firstly describes the employed datasets in Section 2.1. Secondly, the EGM analysis pipeline is defined, consisting the generation of synthetic tracings (Section 2.2), the DL architecture (Section 2.3) and the list of performed experiments (Section 2.5).

### 2.1 Materials

A proprietary EGM delineation dataset was developed in the Hospital Universitario Virgen del Rocío (Sevilla, Spain). This dataset comprises 312 EGM recordings of variable size taken from 77 patients who underwent AP or AVRT ablation, following the ablation protocol recommended in the standard-of-care. The LF and FF activations were manually annotated using a Python tool to mark their onsets and offsets, and these fiducials were then validated by a panel of certified cardiologists. A LF activation was considered when the catheter was placed into a specific anatomical structure (e.g., the left ventricle) and the EGM depicted a high-frequency activation, whereas the FF activation was considered a low-frequency activation occurring elsewhere but propagated to the local tissue (e.g., atrial activation in the left ventricule). In total, 20,671 LF, 13,354 FF and 318 stimulation artifacts annotations were generated. All interventions recorded 5 bipolar EGMs from decapolar catheter (CS-1 or proximal through CS-5 or distal) during pacing or application of extrastimuli while testing for decremental conduction. A Bard Labsystem Pro EP Recording System ⓒ was used (1,000 Hz sampling frequency, 16 bits resolution, 2.5 *μ*V/bit, bandpass-filtered in [30, 500] Hz).

**FIGURE 1:**
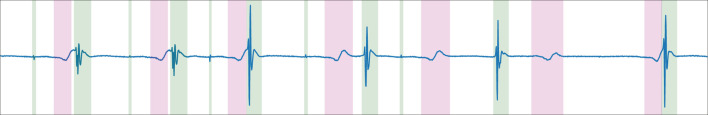
Generated ground truth for an intracavitary electrocardiographic recording at the coronary sinus. The green and magenta overlays represent, respectively, local field activations from the coronary sinus and the ventricular far field. The recording presents ventricular pacing and decremental properties.

The annotations were represented as binary masks for their usage as optimization targets in the segmentation architectures, where a mask of shape {0,1}^3×*N*^ was *True*-valued whenever a specific sample *n* ∈ [0, *N*] was contained within a stimulation, LF or FF activation (indices 0, 1 and 2, respectively) Jimenez-Perez et al. (2021b). The dataset was split 75%–25% so that all bipolar EGMs from the same patient would either be in the training or the testing sets, producing a training set and a held-out testing set (49 and 28 patients, respectively). Figure 1 shows an annotated EGM signal.

A second, completely separate dataset was annotated for clinical validation and was not used for model training or validation. This dataset did not contain delineation annotations (onsets/offsets of LF and FF activations), but clinical labels associated to EGM fragments in which decremental conduction was elucidated. The study protocol consisted in the application of a simple pacing (S = [400, 600] ms) followed by an extra-stimulus (S_2_ = effective refractory period (ERP) + [20, 60] ms), measuring the delay in response caused by the AV node. The recordings were annotated by expert electrophysiologists, where three possible labels were assigned to each recording: decremental (if the time delay after S_2_ exceeded 10 ms), non-decremental or non-interpretable (loss of capture in S_2_ or no conduction through AV node). In total, 321 recordings from 50 patients were annotated and analysed.

### 2.2 Synthetic data augmentation

**FIGURE 2:**
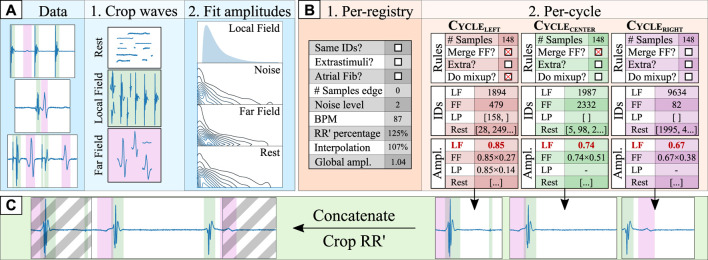
Synthetic data generation pipeline. The data pre-processing step [**(A)**; blue shading] consists of: (1) cropping the ground truth segments into different data “pools” (local field [LF] in green, far field [FF] in magenta, and rest); and (2) fitting the original segment amplitudes to log-normal distributions with respect to the amplitude of the local field (*amplitude*_segment_/*amplitude*_*LF*_). The cycle orchestration step [**(B)**; orange shading] involves: (1) generating a set of registry-wide rules for all cardiac cycles; and (2), generating a set of per-cycle rules (e.g., merging the FF component with the LF), retrieving the specific segment croppings and computing the segment amplitudes for the left, central and right cardiac cycles. Finally, in the synthetic composition step [**(C)**; green shading], the three cardiac cycles are independently generated by firstly generating a baseline of rest segments of sufficient size and adding over it the drawn segments. Then, they are concatenated into a synthetic trace and cropped into a single (central) cardiac cycle, discarding the grayed area.

EGM recordings have segments of electrical silence (or rest), in which one or several LF or FF activations may be contained. Taking advantage of this modular structure, an algorithm for generating synthetic data was developed in this work. The algorithm has two major steps: data pre-processing and trace generation. Figure 2 schematically represents the synthetic data augmentation pipeline.

#### 2.2.1 Data pre-processing

The data pre-processing step consisted in two phases. In the first phase, the annotated ground truth was cropped in its fundamental segments, separating into independent “sets of segments” the LF, FF, LP, stimulation and rest segments. The FF and rest segments were low-pass filtered (100 Hz, 2nd order Butterworth filter) to suppress any unannotated LP in its trace. Moreover, each segment was onset/offset corrected so its voltage started and ended in zero for easier synthetic composition. Finally, the LF morphologies were subdivided into LF and LP morphologies according to whether the segment displayed a length shorter than 25 samples as a rule of thumb.

**FIGURE 3:**
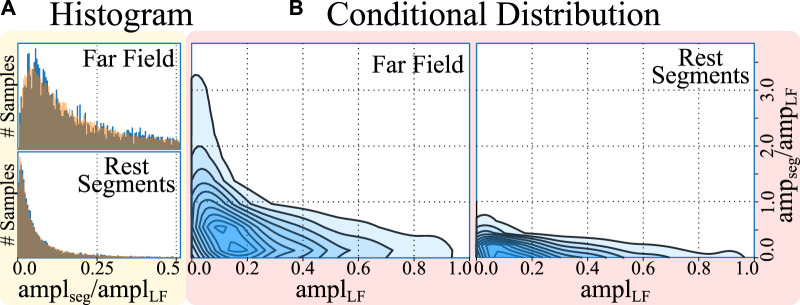
Histogram **(A)** and conditional distribution **(B)** of amplitudes (amp) of the cropped far field (FF) and rest segments with respect to the amplitude of the local field (LF). The histograms represent, in blue, the amplitudes of the segments and, overlaid in orange, the samples drawn from a log-normal distribution, demonstrating a good fit. The conditional distribution represents the kernel density estimates of the relative segment amplitude (*y*-axis) with respect to the LF amplitude of the cardiac cycle (*x*-axis), demonstrating larger segment amplitude at smaller local field amplitudes.

In the second phase, the segment’s morphology was separated from its voltage by modelling its amplitude. Given that the amplitude profile of each segment (*amplitude*_segment_) has a strong dependence with the amplitude of the LF component (*amplitude*_LF_; see Figure 3B), the segment amplitudes could not be fit in a single distribution. For this purpose, firstly, the amplitude of the LF was split into 10 bins (dividing the [0,100]% amplitude interval in increments of 10%). Secondly, for each LF amplitude bin, a log-normal distribution was fitted to model the amplitude distributions of the sub-set of FF and rest segments that accompanied each specific LF fragment, totalling 10 log-normal distributions per segment type. Finally, the amplitude of the LF and LP segments were fitted independently of the amplitude of any other fiducial, with log-normal distributions as well. Once the amplitudes had been fitted, all segments in the “segment pools” were normalized to their maximum absolute value (“max abs” scaling).

#### 2.2.2 Synthetic trace generation

The synthetic trace generation step aimed at producing bipolar EGM signals corresponding to a single cardiac cycle at a time. The resulting synthetic traces were intentionally crafted to deviate from strict physiological replication, in accordance with our clinical collaborators. This design decision was made because of the constraints posed by the size of the development dataset, which was comprised of few samples with manual annotations, which severely hindered the model’s coverage of the real data distribution when used “as-is” for model training. In consequence, the generated synthetic traces intentionally cover iECG morphologies much beyond the ones found in the development set, by composing traces with pseudo-randomly located far field, local field, extrastimuli and DEEP activations.

Synthetic generation consisted of two steps. The first step revolved around probabilistically generating per-registry and per-cycle rules (see Figure 2B). Per-registry rules governed conditions that affect all cardiac cycles within a registry, altering how the per-cycle rules were generated. To produce signals that are robust to QRS segmentation errors or to any physiological differences in LF/FF locations, three cardiac cycles were generated for each patient, which were then cropped to preserve the context of the central cardiac cycle (see Figure 2C). Some examples of per-registry rules are the percentage of the left- and right-most cycles that is preserved, whether all cardiac cycles in a registry have the same morphology, or whether the registry contains stimulation artifacts. Per-cycle rules, on their behalf, governed conditions that affect a single cardiac cycle. For this purpose, different segments (LFs, FFs, LPs and rest segments) and their respective amplitudes were drawn from the sets of segments and amplitude distributions for each cardiac cycle. Given a pre-defined probability, some segments might not be drawn for a specific cardiac cycle (e.g., in the case of AV block, no ventricular activation might take place). If the “same morphology” boolean was toggled, the same segments were drawn for all cardiac cycles, although the amplitudes might vary. Finally, each segment positioned in some location ([0,100]%) of its corresponding cardiac cycle. A full description of the per-registry and per-cycle rules is reported in the Supplementary Materials.

After generating the per-registry and per-cycle rules, the final synthetic trace was composed. Firstly, the rest segments were multiplied by their respective amplitudes and concatenated to form a baseline upon which to place the rest of the segments. Then, each drawn segment (LFs, FFs and LPs) was multiplied by its amplitude and placed in the trace by adding it to the baseline, starting at a specific index, placing them spatially into the registry. These indices were kept in memory to generate the ground truth of the delineation, indicating the precise onset and offset of each segment. To maximize variability, each segment was given a chance to be interpolated to 75%–125% its original length and a chance to be merged with another waveform using Mixup Zhang et al. (2018), a data augmentation strategy that produces a linear combination of different segments. Finally, once all segments were added into the baseline, the noise and baseline wander were added to the trace and the final segment was cropped according to the “RR’ percentage” generated in the global conditions. Figure 4 in the Supplementary Material provides some examples of real and synthetic electrogram signals and traces, respectively. Differences can be observed in the figure between real and synthetic iECG data. However, the synthetically generated traces were not designed to serve as physiological replicas of real data, but to extend the limited original dataset to cover the large variability of iECG signals due to the characteristics of the acquisition and the underlying arrhyhthmia required to improve the training of segmentation models.

**FIGURE 4:**
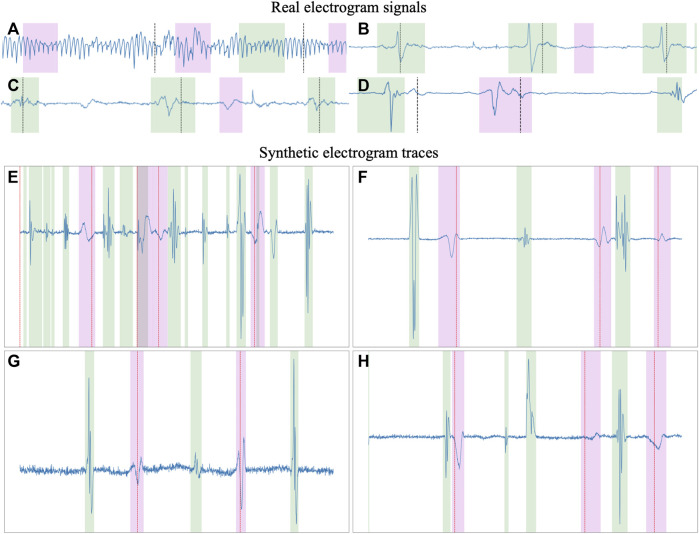
Examples of real **(A–D)** and synthetic **(E–H)** electrogram signals and traces, respectively. The green and magenta overlays represent the local and far field activations, respectively. Substantial differences can be observed between real signals and synthetic traces, the latest providing a larger variability in signal characteristics, making them more suitable for training segmentation models than a limited dataset of clinically-obtained electrogram data.

### 2.3 Architecture

The U-Net Ronneberger et al. (2015) is a state-of-the-art convolutional neural network (CNN) that is organized as an encoder-decoder structure and is usually employed in medical imaging segmentation tasks. The encoder-decoder is a type of artificial neural network (ANN) topology revolving around the usage of an encoder for obtaining highly abstract data representations (usually tied to reducing input complexity), and a decoder to leverage the abstracted information into a specific output LeCun et al. (2015). In the case of the U-Net, the encoder and the decoder are conformed of convolutional operations, which act similarly to trainable digital filters and emphasize local relationships in data (either spatial or temporal, depending on the data to be analyzed), and pooling/upsampling operations, which allow models to train filters over more distant elements of the input image by reducing/increasing tensor size. Finally, the encoder and the decoder are connected by “skip connections”, which recover the input information at different levels of abstraction for: a) defining segmentation borders in a more precise manner, which could be lost with the pooling layers; and b) preventing problems arising from vanishing gradients when optimizing the model’s weights Ronneberger et al. (2015). The number of trainable convolutional filters is usually doubled after every pooling operation and halved after every upsampling operation.

Many U-Net-based alternatives exist due to its high performance for a variety of tasks Litjens et al. (2017). Most works explore altering the model’s original design decisions, such as the number of convolutional operations before any pooling operation (hereinafter, model “width”), the number of times the model reduces the input size (model “depth”), number of convolutional filters, employed non-linearity or choice of regularization Jimenez-Perez et al. (2021b). Some authors have even developed heuristics for automatically adjusting the model’s training parameters and reducing the developer’s workload Isensee et al. (2021). Other authors have attempted at incorporating state-of-the-art additions such as self-attention mechanisms Vaswani et al. (2017), which allow the weights of an operation to be controlled by a secondary set of weights, effectively controlling feature importance Prabhakararao and Dandapat (2020). While some adaptations of attention mechanisms exist for convolutional operations, this work explores the application of efficient channel attention (ECA) due to its low computational overhead Wang et al. (2020).

**FIGURE 5:**
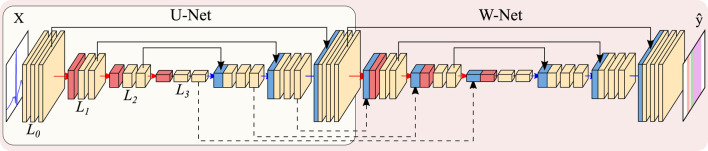
Representation of the U-Net (encircled in yellow) and W-Net architectures (encircled in red, containing the U-Net). Both networks are instantiated with 3 levels and 2 convolutional blocks per level. Arrows represent operations, while blocks are indicative of output tensors. Convolutional filters are doubled at each level, so that level *L*_*i*_ has 2^*i*^ *N* channels per level (with N being the starting number of channels), whereas pooling and upsampling have a kernel size of 2. Color code: convolutions (yellow), pooling operations (red), upsampling operations (blue), concatenation operations (black).

Other works explore topological changes, either by embedding the U-Net into another structure Xia and Kulis (2017); Chen et al. (2018) or by increasing its connectivity (number of times the output tensors from each convolutional operation are used) Zeng et al. (2019). In this work, the W-Net architecture Xia and Kulis (2017) was employed given its good performance in other segmentation domains, such as the segmentation of echocardiographic images Xu et al. (2020). The W-Net involves using two U-Nets, where the second network takes as input the output of the first network, and employ “skip connections” not only between each encoder/decoder pair but also between the decoder of the first U-Net and the encoder of the second. This second U-Net increases the model’s capacity, which is usually tied to better performing models. A visual representation of the U-Net and the W-Net are presented in Figure 5.

### 2.4 Model evaluation

**FIGURE 6:**
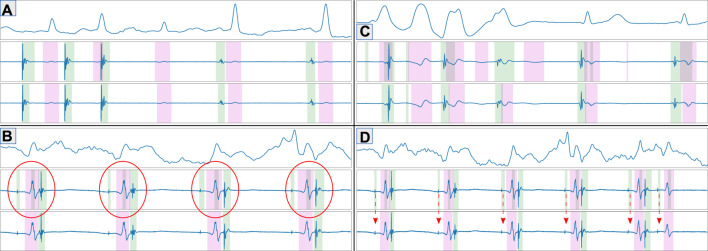
Representative examples of model predictions depicting good examples **(A)**, prediction errors caused by higher sensitivity than ground truth [**(B)**; high-frequency component within the far field], true prediction errors **(C)** and errors attributable to wrongly annotated ground truth **(D)**. The figures show the ECG reference (top), predicted fiducials (middle) and ground truth (bottom). Green and magenta regions represent local and far field components, respectively.

The model’s performance was calculated in two ways: by evaluating the performance using typical delineation metrics; and by addressing the precision in a clinical validation dataset. Firstly, detection and delineation metrics were computed with respect to the ground truth. Detection metrics measured localized matches with the ground truth (i.e., segments occurring at the same time in the prediction and the ground truth). Delineation metrics, on their behalf, measured error at the localization of the segment’s onset and offset with respect to the reference. The detection and delineation metrics were computed before and after filtering: given the large number of LPs detected within the confines of FF activations (see Section 3 and Figure 6B), a secondary set of metrics was computed, consisting in measuring the aforementioned detection and delineation metrics, but avoiding counting these as false positives.

**FIGURE 7:**
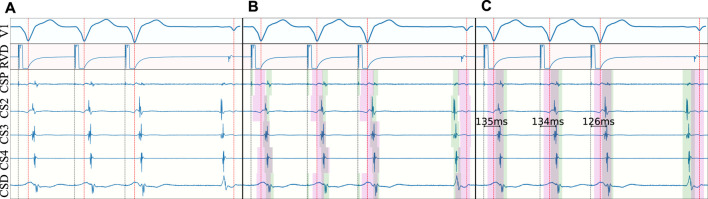
Decrement computation algorithm on a non-decremental trace. In **(A)**, the surface ECG is delineated (red dotted line) and the stimulation onset is located (gray dotted line). In **(B)**, the detected QRS’ are employed to locate the onsets and offsets of the local field (green) and far field (magenta) activations for each bipolar electrode. In **(C)**, majority voting is performed to obtain a single set of onsets/offsets for all electrodes, the predictions are cleaned (e.g., spikes related to pacing) and the measurements are produced.

With respect to the clinical validation metrics, sensitivity and specificity figures are reported for the accurate detection of decremental response in the annotated registries. For producing a prediction, five stages were followed. Firstly, the QRS complex was firstly detected using the delineator proposed in Jimenez-Perez et al. (2021a). Secondly, the EGMs of each cardiac cycle were independently predicted, obtaining the onsets and offsets of each segment for each lead. Thirdly, a single onset-offset pair was selected across all leads by majority voting. This was useful for this specific clinical problem, given that the spatial configuration of the employed catheter allowed for certain synchronicity across leads (see Section 2.1). In fourth place, a matching algorithm was employed to tie each stimuli to its response. For this purpose, the origin of the stimulation was firstly located (*stim*; auricular or ventricular origin) for, then, determining the delay to the response (*resp*; ventricular or auricular response, respectively). In this step, a series of exceptions were defined (e.g., uncoordinated stimulation-response, too distant response, absence of response or too different response morphology, among others), which lead to the exclusion of the excerpt for its posterior analysis. In fifth and final place, the distances between the stimulus and the response (Δ*t*_*i*_ = *resp*_*i*_ − *stim*_*i*_) were computed. Given the stimulation protocol (single pacing followed by extrastimulus S_2_), the delay Δ*t* between the two last stimuli (Δ*t*_*N*−1_ and Δ*t*_*N*_, respectively) was measured and decremental response was considered if (Δ*t*_*N*−1_ − Δ*t*_*N*−1_) > 10 *ms*. The final value was corrected with the lag of the highest cross-correlation between the last two responses. Figure 7 depicts the decrement computation algorithm on a sample EGM.

### 2.5 Experiments

Model performance was assessed by training several model topologies, isolating specific changes to test the contribution of each element in the model. Firstly, the best architectural configuration was assessed by comparing the performance of the U-Net and W-Net (for depths 5 and 6, independently), both with and without ECA. Secondly, the effect of using a pre-trained model for the task of ECG delineation was tested, taking the weights from a model for ECG delineation Jimenez-Perez et al. (2019), Jimenez-Perez et al. (2021a), Jimenez-Perez et al. (2021b). Finally, the effect of applying a loss function that forces higher sensitivity was explored by doubling the executions, comprising training models with and without the loss function. The loss function employed the edge detector described in Jimenez-Perez et al. (2021a) for computing the true positives (TP), false positives (FP) and false negatives (FN), which were in turn employed for computing the classic sensitivity score: *Se* (%) = *TP*/(*TP* + *FN*).

Some aspects were kept constant throughout all experiments. On the one hand, the application of some regularization strategies such as SDr or certain types of DA was associated with better performance, so these were always applied. A random seed (123456) was employed for reproducibility, the Adam optimizer was used Kingma and Ba (2014), leaky ReLUs Xu et al. (2015) were selected as the non-linearities of choice, and the number of base channels was kept the same (32, doubled/halved on the pooling/upsampling operations). Due to limitations in the completeness of the annotated ground truth (see Figure 6D), training was solely performed using synthetic data. However, as reported in Jimenez-Perez et al. (2021a), this was associated with only a slight decrease in performance as compared to using synthetic and real data, and outperformed training the model only with real data. All executions were performed with a NVIDIA Titan Xp GPU using PyTorch.

## 3 Results

The best performing model was a W-Net model with 6 levels, optimized solely with the Dice loss. The model obtained precisions of 76.44%, 74.73% and of 100.0%, and recalls of 94.84%, 95.23% and 100.0% for localizing LF activations, FF activations and extrastimuli, respectively. The model also attained an average delineation error of 4.20 ± 13.89 and −6.45 ± 19.86 ms when localizing the LF’s onsets and offsets, respectively; and of 3.74 ± 19.26 and −5.71 ± 21.91 ms when estimating the onsets and offsets of the FF. The localization of stimulations was very precise, with onset errors of −0.68 ± 1.27 ms. Given the ambiguity between some segments and the errors in the dataset annotations (as it will be discussed in Section 4), a metric was obtained by merging the binary masks of LF and FF components, which obtained a precision, recall, onset and offset errors of 90.02, 97.53, 83.52, 9.04 ± 26.09 and −10.65 ± 29.32, respectively. A detailed description of the per-wave metrics of the model (precision, recall, Dice score, onset error and offset error) is reported in Table 1.

**TABLE 1:** Precision (%), recall (%), Dice score (%), onset error (mean [M] ± standard deviation [SD], in miliseconds) and offset errors (M ± SD, in miliseconds) of our best performing model.

	Precision (%)	Recall (%)	Dice (%)	Onset error (M ± SD)	Offset error (M ± SD)
Local Field	76.44	94.84	77.37	4.20 ± 13.89	−6.45 ± 19.86
Far Field	74.73	95.23	73.22	3.74 ± 19.26	−5.71 ± 21.91
Local + Far Field	90.02	97.53	83.52	9.04 ± 26.09	−10.65 ± 29.32
Stimulation	100.0	100.0	94.78	−0.68 ± 1.27	-
Local Field (≤25 ms)	75.04	67.98	45.41	1.51 ± 1.41	−5.69 ± 2.91
Local Field ( > 25 ms)	80.77	96.18	78.68	4.04 ± 13.42	−3.65 ± 16.67

A secondary set of measurements was computed by discarding as false positives any LF that occurred within the confines of a FF, as described in Section 2.5. With this secondary metric, the model obtained precisions of 91.28%, 77.78% and of 100.0%, and recalls of 94.86%, 95.25% and 100.0% for localizing LF activations, FF activations and extrastimuli, respectively. The model had an average delineation error of 3.89 ± 14.56 and −6.16 ± 20.25 ms when localizing the LF’s onsets and offsets, respectively; and of 3.47 ± 20.03 and −5.44 ± 22.82 ms in the FF. A more in-depth report of the per-wave metrics of the model is reported in Table 2. Furthermore, some representative examples of the best performing model’s performance have been plotted in Figure 6. To aid in the discussion, the samples were grouped according to the different types of errors produced by the network (or absence of). These can be divided into four main categories: good samples (Figure 6A), errors due to increased model sensitivity with respect to the ground truth (Figure 6B), true network errors (Figure 6C), and annotation errors in the database (Figure 6D). Together with the real and synthetic examples depicted in Figure 4 in the Supplementary Material, these results demonstrate the appropriateness of training a segmentation model with a synthetic dataset including a large variability of characteristics, despite the obvious differences in signal morphology with real data, which can only be available in a limited number of settings.

**TABLE 2:** Precision (%), recall (%), Dice score (%), onset error (mean [M] ± standard deviation [SD], in miliseconds) and offset errors (M ± SD, in miliseconds) of our best performing model after discarding small local field activations contained within far field activations.

	Precision (%)	Recall (%)	Dice (%)	Onset error (M ± SD)	Offset error (M ± SD)
Local Field	91.28	94.86	77.37	3.89 ± 14.56	−6.16 ± 20.25
Far Field	77.78	95.25	73.22	3.47 ± 20.03	−5.44 ± 22.82
Local Field + Far Field	91.39	97.57	83.52	7.85 ± 28.52	−9.67 ± 31.77
Stimulation	100.0	100.0	94.78	−0.68 ± 1.27	-
Local Field (≤25 ms)	94.53	67.98	45.41	1.51 ± 1.41	−5.69 ± 2.91
Local Field ( > 25 ms)	94.06	96.19	78.68	4.0 ± 13.51	−3.6 ± 16.76

### 3.1 Model additions

**FIGURE 8:**
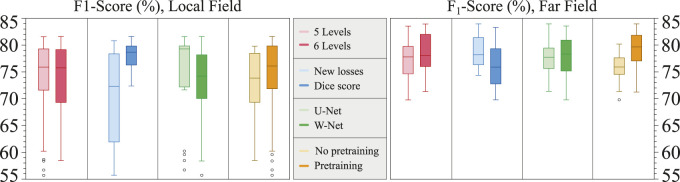
Boxplots of the contributions of the different model additions to the overall model performance, divided into the local field (left) and far field (right). *Y*-axis corresponds to the *F*_1_ score.

The only model addition that showed consistently better results with respect to the baseline was the application of increased model capacity (either with W-Net or with more model depth) and pre-training the model with weights from an ECG delineation model Jimenez-Perez et al. (2021a). Other effects, such as the addition of custom data losses, were generally detrimental for model performance. Figure 8 summarizes the effect of the different model additions.

### 3.2 Clinical validation

The clinical validation model demonstrated good overall agreement with respect to the evaluation of decremental properties. Out of the 321 recordings employed for evaluation of decremental response, 81 (25.23%) were automatically excluded by the rule-based algorithm. When compared to the exclusion criteria proposed by electrophysiologists, the exclusion step reached a sensitivity of 87.06% and a specificity of 97.03%. Out of the 240 remaining, 180 (75%) were evaluated to be decremental and 60 as non-decremental (94.42% accuracy, 96.77% sensitivity, 95.24% specificity). The selected model is not computationally expensive, producing a prediction in 18.9 ± 0.22 ms on GPU (NVidia GeForce GTX 1050 Ti), which is bound to be faster with more modern hardware.

## 4 Discussion

Electrogram segmentation is a crucial task for advancing in the automatization of EP procedures. Currently, physicians must manually produce basic measurements when performing interventions such as AVRT or AP ablation for determining decremental properties or to measure basic intervals. Despite its importance, even state-of-the-art EAM systems only perform basic detection of the most salient wave within a cardiac cycle for computing derived clinical indicators. The inability of performing full signal delineation is limiting, as recent developments in diagnostic markers for catheter ablation such as decrement-evoked potentials are detected through the analysis of portions of myocardial tissue that produce LFs or LPs that are delayed with respect to previous cardiac cycles.

The work presented here builds upon the existing detection and delineation literature by advancing towards an all-purpose iECG analysis system. Similarly to the approach proposed in Jimenez-Perez et al. (2021a), a DL model was trained for automatic data quantification; focusing on quantification counterbalances the drawbacks of DL algorithms with an application that is immediately interpretable by the operator. Given the lack of large-scale iECG datasets annotated for delineation, two main design decisions were made. Firstly, the model was trained solely with synthetic data from a modest dataset of 312 iECG recordings from 77 distinct patients, with ground truth generated for localizing independent LF and FF activations. This synthetic dataset greatly improves model performance in scenarios where data is scarce, and has been proven to be more performant than training on real samples if the data is scarce Jimenez-Perez et al. (2021a). Secondly, the prediction pipeline was designed to analyse excerpts of individual cardiac cycles, whose window of interest was localized with the QRS complex’s barycenter in the surface ECG using a DL model Jimenez-Perez et al. (2021a). Cropping the iECG recordings into individual cardiac cycles allowed the model to adjust the prediction of a specific waveform according to whether the LFs (high frequency components) occurred before or during ventricular depolarization. The combination of these design decisions allowed to alleviate the main limitations found in initial approaches, producing more versatile networks.

Although many models and model additions were explored for pushing performance, model performance seemed to respond similarly to the explored changes (Figure 8). Moreover, the trained models swored a high variance overall in *F*_1_ score, and neither changing model capacity (5 or 6 U-Net/W-Net levels), changing the loss functions (Dice score or new losses) or changing the base architecture (U-Net or W-Net) seemed to significantly improve performance. The only clear improvement in both LF and FF *F*_1_ scores seemed to be starting the training from a model pretrained with an ECG delineation task Jimenez-Perez et al. (2021a), which is consistent to the recent advancements in Self-Supervised pretraining of Computer Vision models (Caron et al., 2021). We hypothesize that one of the factors that cause this variance is the need to add more training data, which is also hinted by the high amount of runs that did not produce a model that consistently converged (i.e., *F*_1_ scores neighbouring 60%).

**FIGURE 9:**
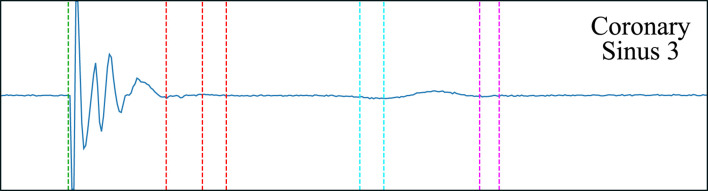
The smoothness of the wave complicates the definition of the local field’s offset (red dashed line) and the far field’s onset and offset (cyan and magenta dashed lines, respectively). Multiple possible onsets/offsets are marked.

The best performing model demonstrated high sensitivity but moderate precision (around 95% and 75%, respectively, for both LF and FF activations in a held-out test set). With respect to the onset/offset localization, the models provided a good fit with respect to the reference (errors of 3.89 ± 14.56 and −6.16 ± 20.25 ms when estimating the LF’s onsets and offsets, respectively; and of 3.47 ± 20.03 and −5.44 ± 22.82 ms at the FF components). Comparing the proposed approach to the existing literature gives the impression of a reduced algorithm performance: some methods reach precision and recall figures nearing 100% Osorio et al. (2017); Felix et al. (2015) and half the SD in onset/offset localization Alcaine et al. (2014). This, however, is misleading for several reasons. Firstly, existing algorithms are only concerned with locating a single LF activation for each cardiac cycle and disregard any other type of activation (e.g., LP or FF), which prevents direct comparison between methodologies. Secondly, all development datasets are private, preventing a fair comparison of methods; the dataset collected for this work consists of real clinical data, making no compromises with respect to signal quality or difficulty. Thirdly, models that are more sensible than specific were sought for, and distinguishing subtle LPs from noise is a challenging task. Finally, the larger delineation errors are to be expected given smoothness at signal initiation and termination (see Figure 9) and the lack of an unified criterion for their definition. Despite the comparatively reduced detection and delineation metrics, the overall performance at locating specific components has proved excellent for a downstream clinical application for the detection of decremental response in AP or AVRT procedures. The model, with a relatively simple post-processing, allowed for the identification of decremental response (Δ*t* > 10 *ms*) with high precision and accuracy, reaching sensitivity and specificity figures of 96.77% and 95.24% specificity, respectively.

The proposed approach has two main advantages. Most importantly, a full delineation of all important iECG fiducials in the registry is performed, as opposed to the localization of the most salient component Osorio et al. (2017); Felix et al. (2015); Alcaine et al. (2014). This is of capital importance at advancing processing for EP procedures and reducing intervention times, as many diagnosis procedures are performed by comparing segments or LP in subsequent cardiac cycles. Additionally, using a synthetic data generation algorithm allows to better control the conditions for predicting a local component, which is highly beneficial: the low specificity reported in Table 1 results from lowering the threshold at which a perturbation can be recognized as a local component (see Figure 6B). Thus, the system is able to propose low intensity, high frequency deflections as candidate local components, which would be too costly and time-consuming to annotated while not necessarily erroneous. The difference between the ground truth and the predictions might represent a limitation of the ground truth rather than of the developed model.

The primary objective of the synthetic data generation was not to be used as realistic data for clinical practice, but to force the model to identify specific iEGM components such as local field, far field, and DEEP signals, including possible changes in signal acquisition (e.g., different type of catheters). The resulting intentional deviation from strict physiological replication proved beneficial, even at a slight loss of realism. This tradeoff between variability and realism in the generated signals is not significantly different from usual data augmentation strategies found in the deep learning literature, in which extreme transformations over the base image are performed but not necessarily evaluated for realism (e.g., the recent GIN-IPA data augmentation technique (Ouyang et al., 2023)). In consequence, it is not straightforward to make a direct comparison between real and synthetic data using similarity-based metrics (e.g., cross-correlation). However, the developed segmentation and classification models were trained exclusively on synthetic data, their accuracy on held-out datasets of real data being a very strong indirect evaluation of the usefulness of the synthetic generation pipeline.

The proposed approach has, however, some limitations that are unique to EGMs as opposed to other cardiac signals such as the ECG. Firstly, expressing the ground truth as a binary mask delimiting each local component, as is performed in this work, might clash with some scenarios where the individual local components should not be merged, giving rise to difficulties when analyzing highly fractionated potentials, where predicting a continuous *True*-valued binary mask spanning the whole fractionation might not be useful for posterior analyses. Secondly, a compromise with respect to the architectural choice might be of need, as the model prediction time is larger than the sampling frequency (7.88 ms per cardiac cycle and lead). This, however, might be circumvented by good implementation in an EAM platform, by multi-threading, processing the iECG while the catheter changes position or the system waits for respiration cues or by providing the outputs with a slight delay. Thirdly, the model could not be trained leveraging real data, partially due to the necessity to improve the quality of the ground truth annotations: many waves were not correctly delineated and accounted for false positives (Figure 6D), requiring re-annotation, and more prevalence of fractionated potentials is needed to assess the generalizability of our approach. Finally, the developed rules for the synthetic DA algorithm allow for much higher complexity, requiring the inclusion of more real-world casuistry to enhance performance.

## 5 Conclusion

The proposed methodology for the analysis of iECG recordings has proven to be useful in other signal analysis tasks such as ECG delineation Jimenez-Perez et al. (2021a), hinting at the feasibility of a good-performing, all-purpose EGM annotation tool. Current results show great promise while being, to the best of our knowledge, the first tool in the literature allowing the delineation of all local components present in a recording. The algorithm, based on an encoder-decoder DL architecture, was trained solely with synthetic data according to a rule-based algorithm that allows for controlling the generation process. The algorithm is, however, faced with several limitations in the dataset, data generation and data representation. Nevertheless, the development of an all-purpose EGM delineation model is a key tool for unlocking a wide array of downstream tasks, ranging from the automatic identification of myocardial portions of scar presenting DEEPs to the exploration of morphological indicators that might aid in diagnosis or risk stratification.

## Data Availability

The datasets presented in this article are not readily available because Patient data is not available outside of the hospital. Requests to access the datasets should be directed to juan.acostamartinez@gmail.com.
