# Correction: Delineation of intracavitary electrograms for the automatic quantification of decrement-evoked potentials in the coronary sinus with deep-learning techniques

**DOI:** 10.3389/fphys.2025.1726050

**Published:** 2025-11-10

**Authors:** Guillermo Jimenez-Perez, Juan Acosta, Álvaro José Bocanegra Pérez, Eduardo Arana-Rueda, Manuel Frutos-López, Juan A. Sánchez-Brotons, Helena Llamas-Gómez, Rodrigo Di Massa Pezzutti, Carmen González de la Portilla Concha, Oscar Camara, Alonso Pedrote

**Affiliations:** 1 PhySense Research Group, BCN MedTech, Universitat Pompeu Fabra, Barcelona, Spain; 2 Arrhythmia Unit, Department of Cardiology at Virgen Del Rocío University Hospital, Sevilla, Spain

**Keywords:** intracavitary electrograms, decrement-evoked potentials, deep-learning, automatic signal delineation, coronary sinus, local field components, synthetic data

In the published article, an **author name** was incorrectly written as [Helena Llamas-López]. The correct spelling is [Helena Llamas-Gómez].

The original article has been updated.

